# Hypo-osmotic stress is an anticipatory trigger of heat-resistance in presumptive extraintestinal pathogenic *Escherichia coli* isolated from treated sewage

**DOI:** 10.3389/fmicb.2025.1676613

**Published:** 2025-10-08

**Authors:** Kanghee Ryu, Daniel Yu, Paul Stothard, Simon J. G. Otto, Michael Gänzle, Norma J. Ruecker, Norman F. Neumann

**Affiliations:** ^1^School of Public Health, University of Alberta, Edmonton, AB, Canada; ^2^Department of Agricultural, Food and Nutritional Science, University of Alberta, Edmonton, AB, Canada; ^3^HEAT-AMR Research Group, School of Public Health, University of Alberta, Edmonton, AB, Canada; ^4^Centre for Healthy Communities, School of Public Health, University of Alberta, Edmonton, AB, Canada; ^5^Antimicrobial Resistance – One Health Consortium, Calgary, AB, Canada; ^6^Water Quality and Regulatory Assurance, Calgary, AB, Canada

**Keywords:** extra-intestinal pathogenic *Escherichia coli*, ExPEC, disinfection, heat-resistance, osmotic stress, stress resistance, sewage treatment, wastewater treatment

## Abstract

Extraintestinal pathogenic *Escherichia coli* (ExPEC) are responsible for a variety of human infections (urinary tract infections, septicemia, meningitis) and can be routinely isolated from treated sewage. However, the phenotypic properties mediating treatment resistance in ExPEC remain elusive. Herein, we examined heat-resistance in presumptive wastewater ExPEC (W-ExPEC) surviving sewage chlorination or full-scale wastewater treatment. Interestingly, heat-resistance in W-ExPEC was triggered by exposure to hypo-osmotic conditions (i.e., sterile distilled water), resulting in a 10- to 1,000-fold increase in heat-resistance compared to cells exposed to iso-osmotic conditions (i.e., phosphate buffered saline). Remarkably, hypo-osmotic induction of heat resistance occurred extremely fast, in as little as 30 s, and was reversible, demonstrating the phenotypic plasticity of this stress response. Hypo-osmotic stressed W-ExPEC strains survived 58 °C temperatures for up to 20 min – this compared to the clinical reference ExPEC strain, CTF073, which became non-culturable after only 5 min of exposure at this temperature (>8 log10 decline in culturability). The upper thermotolerance level of W-ExPEC (defined as the temperature where culturability was lost after 5 min of exposure) was 62 °C, compared to 58 °C for CFT073. The finding that osmotic stress acts as an anticipatory inducer of heat resistance in W-ExPEC is novel, providing some insights into the possible mechanisms triggering a treatment resistant phenotype in W-ExPEC. The evolution of treatment resistance is worrying prospect for public health, given that waste treatment is a hallmark of infectious disease control in modern society.

## 1 Introduction

Microbes are incredibly adaptable and have a remarkable ability to tolerate a wide range of normally lethal stressors. Certain strains of *Escherichia coli* appear to have adapted to live and survive in sewage treatment plants (Zhi et al., 2016; Zhi et al., 2017; Zhi et al., 2019) – facilities specifically engineered to destroy these microbes. The potential for microorganisms to evolve resistance to waste treatment is a concerning prospect for public health given that sewage treatment is a hallmark of modern sanitation.

As a species, *E. coli* possesses a myriad of adaptive stress response mechanisms, including the universal stress response, SOS response, oxidant tolerance (ROS) response, various efflux and toxin-antitoxin systems (Trastoy et al., 2018), and the general stress response (*rpoS*-mediated), which alone is estimated to involve at least 10% of all genes in the *E. coli* genome (Landini et al., 2014). Interestingly, many of these resistance mechanisms confer resistance against specific stressors specifically encountered during the sewage treatment process, including chlorination, osmotic stress, microbial predation/competition, nutrient deprivation, temperature shock, heat (e.g., during the composting of biosolids), and oxidant- and UV-mediated disinfection. For instance, several chlorine-sensitive transcription factors have been characterized, including *yjiE* (Gebendorfer et al., 2012), *nemR* (Gray et al., 2013), *rclR* (Parker et al., 2013), and *hypT* (Drazic et al., 2013), in addition to transcription factors associated with oxidative and UV stress, such as *oxyR* (Imlay, 2008) and the *soxRS* system (Trastoy et al., 2018). Cross-resistance to chlorination and oxidative stress may also be conferred by other stress resistance strategies in *E. coli*, such as through the production of heat shock proteins (Winter et al., 2005). In addition, Wang et al. (2020) demonstrated that a genetic island known as the transmissible locus of stress tolerance (tLST), previously designated as locus of heat resistance (LHR), encodes various heat shock, oxidative stress, and cell envelope stress proteins (Mercer et al., 2017), which, when possessed, results in multiparameter resistance to chlorine, heat, and other oxidants.

Wastewater treatment, therefore, could represent a powerful natural selection force for the evolution of microbes – a phenomenon reflected in the recent discovery of naturalized *E. coli* populations adapted to live and survive within sewage/wastewater treatment plants (Zhi et al., 2016; Zhi et al., 2017; Zhi et al., 2019). Indeed, compared to laboratory reference strains and their enteric counterparts, naturalized wastewater strains appear to be enriched in stress-related genes (Yu et al., 2024) and have been shown to be more resistant to chlorine and advanced oxidants, possess an extreme heat-resistant phenotype, display more robust biofilm production, and are selectively enriched across the wastewater treatment process (Zhi et al., 2017; Zhi et al., 2019; Wang et al., 2020; Ma et al., 2021). Concerningly, recent data suggest that the extraintestinal pathogenic *E. coli* (ExPEC), which includes the urinary pathogenic *E. coli* (UPEC), bloodborne [septicemia] *E. coli* (BBEC), and neonatal meningitic *E. coli* (NMEC), also appear to have adapted to survive sewage/wastewater treatment (Zhi et al., 2020; Yu et al., 2022a). Anastasi et al. (2010) and Anastasi et al. (2013) found that certain ExPEC strains were more prevalent at different stages of sewage/wastewater treatment, including chlorination and UV disinfection, and virulence gene screening indicated that up to 59.5% of the isolates surviving treatment carried UPEC-associated virulence genes such *as papAH, papEF, papC, hlyA, cnf1,* and *iroN* (17), and were especially notable after chlorine disinfection (Anastasi et al., 2013). Similarly, Adefisoye and Okoh (2016) found that 41.7% of *E. coli* strains isolated from treated wastewater effluents were possible UPEC and based on virulence gene profiling. Moreover, Calhau et al. (2015) characterized an *E. coli* isolate from hospital wastewater effluent carrying known UPEC virulence genes (*iutA, sfa/foc, papAH*) and pathogenicity islands (PAI IV_536_, PAI I_CFT073_, PAI II_CFT073_) belonging to the ST131 sequence type, the predominant ExPEC pandemic lineage worldwide (Nicolas-Chanoine et al., 2014). Using a comprehensive whole genome comparative approach, Zhi et al. (2020) and Yu et al. (2022a), similarly demonstrated that several *E. coli* isolates recovered from treated wastewater effluents represented clinically-relevant UPEC, BBEC, and NMEC strains. ExPEC strains isolated from chlorinated sewage and full-scale wastewater treated effluents were dominated by clinically relevant sequence types (i.e., ST131, ST95, ST127, and ST640). When compared to clinical ExPEC strains, several of the wastewater isolates shared over 99% whole genome similarity and had the exact same complement of virulence and antibiotic resistance genes with a clinical counterpart, suggesting that *E. coli* strains surviving the wastewater treatment process may possess the capacity to cause extraintestinal disease (Zhi et al., 2020; Yu et al., 2022a). Similarly, Paulshus et al. (2019) observed that ExPEC dominated the population of *E. coli* in treated wastewater, and proposed that either ‘higher-than-expected’ levels of ExPEC infections exist in the human population, or that ExPEC strains naturally occur in wastewater. Collectively, this has led to growing concerns about the role that water might play in the transmission of ExPEC (Graham et al., 2021).

Although we, and others, have previously demonstrated that ExPEC appear to differentially survive wastewater treatment, we have yet to assess their phenotypic responses to treatment stressors. Since heat, chlorine and oxidant resistance in naturalized wastewater *E. coli* strains appear to be mediated by the tLST (Wang et al., 2020), our primary objective was to evaluate the phenotypic patterns of heat-resistance in these wastewater-derived ExPEC strains as a proxy of treatment resistance. Herein, we demonstrate that W-ExPEC strains lack the tLST but still display a strong heat-resistance phenotype – one that is rapidly triggered by exposure to water (within seconds), and which suggests that an alternate and novel cellular mechanism may regulate heat-resistance in these ExPEC strains.

## 2 Materials and methods

### 2.1 Bacterial strains

All *E. coli* strains used in this study are listed in Table 1. The five W-ExPEC strains, WU1036 (BBEC), WU664 (BBEC), 4B8 (BBEC), 2F5 (BBEC), and 3C4 (NMEC), were isolated from chlorine-treated sewage or from treated wastewater effluents as previously described by Zhi et al. (2020), and the strains were genomically characterized by Zhi et al. (2020) and Yu et al. (2022b). *E. coli* ATCC 25922 and MG1655 were used as a heat-susceptible and -intermediate strains, respectively. CFT073 was purchased from the American Type Culture Collection and used as a reference clinical ExPEC strain with low heat resistance. Naturalized wastewater *E. coli* strains WW10 and WW69, originally isolated by Zhi et al. (2016), were previously identified as tLST-positive by Wang et al. (2020) and were used as extreme heat-resistant reference strains. All *E. coli* isolates were stored at -80 °C prior to use in assays described below.

**Table 1:** *Escherichia coli* strains used in this study to assess heat-resistance.

Strain	Description	Reference
ATCC25922	Clinical isolate susceptible to heat	
MG1655	*Escherichia coli* str. K-12 substr. MG1655	
CFT073	Clinical ExPEC reference isolate – UPEC^a^	
WW10	Naturalized wastewater isolate from chlorinated wastewater (tLST-positive)	Zhi et al. (2016)
WW69	Naturalized wastewater isolate from chlorinated wastewater (tLST -positive)	Zhi et al. (2016)
WU1036	ExPEC isolate from chlorinated wastewater (tLST -negative) – BBEC^b^	Yu et al. (2022a)
WU664	ExPEC isolate from chlorinated wastewater (tLST -negative) – BBEC^b^	Yu et al. (2022a)
4B8	ExPEC isolate from UV treated wastewater effluent (tLST -negative) – BBEC^b^	Yu et al. (2022a)
2F5	ExPEC isolate from UV treated wastewater effluent (tLST -negative) – BBEC^b^	Yu et al. (2022a)
3C4	ExPEC isolate from UV treated wastewater effluent (tLST -negative) – NMEC^c^	Yu et al. (2022a)

^a^Urinary pathogenic reference strain of *Escherichia coli*.

^b^Bloodborne or septicemia-causing strain of *Escherichia coli* isolated from wastewater.

^c^Neonatal meningitis-causing strain of *Escherichia coli* isolated from wastewater.

### 2.2 Assessing heat resistance of wastewater ExPEC strains

All *E. coli* isolates were cultured in 3 mL of tryptic soy broth (TSB, BD Difco) at 37 °C and at 200 rpm for 24 h in a shaking incubator (Innova™ 42 Incubator Shakers, New Brunswick Scientific™, Eppendorf). The cultured cells were harvested by centrifugation (9,000 X g for 5 min at room temperature) and were washed twice with phosphate-buffered saline (PBS, HyClone) or autoclaved distilled water. Cell pellets were resuspended and diluted to a final concentration of approximately 1×10^8-9^ most probable number per mL (MPN/mL) in 3 mL of PBS or water.

Since these laboratory manipulations often inadvertently cause stress to cells [i.e., cold stress (setting up experiments at room temperature), nutrient deprivation (suspension in PBS or water)] we first sought to determine what effect these laboratory manipulations had on the survivability of all *E. coli* strains and ensure that observations about heat-resistance were not due to other introduced stressors. In some experiments, samples were incubated under short-term (< 1 h) and long-term (24 h) exposures to multiple environmental stresses including cooler temperature (20 °C), nutrient depletion, and hypo-osmotic stress, and their survivability assessed by culture-based methods (see below). These bacteria were also subsequently exposed to heat-stress under a range of temperatures (25 °C to 66 °C) and for varying lengths of time (0–60 min) [see below].

A modified most probable number (MPN) spot assay was used to enumerate surviving bacterial cells. Each bacterial sample was serially diluted ten-fold with PBS buffer. Five μL of the dilutions were spotted on tryptic soy agar (TSA, BD Difco) plates in triplicate and incubated at 37 °C for 24 h. Depending on the presence or absence of colonies on the spotted areas of TSA plates, the number of positive spotted areas was counted and converted to MPN/mL.

To evaluate heat resistance levels of *E. coli* isolates, 100 μL of a bacterial suspension was dispensed into three wells of a 96-well polymerase chain reaction (PCR) microplate or 0.2 mL PCR tubes and subjected to heat treatment at various temperatures (ranging from 55 °C – 66 °C) and for varying durations (0–60 min), using a thermal cycler (Applied Biosystems® 2,720 Thermal Cycler). The modified MPN method described above was used to estimate surviving bacterial cells after this heat treatment. All experiments were independently repeated at least three times.

### 2.3 Hypo-osmotic induction of heat resistance

Osmotic stress was shown to be a major inducer of the heat-resistant phenotype in our ExPEC strains, and as a result, we sought to determine how quickly this phenotype could be induced in these strains. Isolates were incubated in TSB at 37 °C for 24 h in a shaking incubator at 200 rpm and washed twice with PBS by centrifugation (9,000 X g for 5 min at room temperature). The washed cells were resuspended in PBS to a final concentration of approximately 1×10^9^ MPN/mL. Twenty μL of this resuspended sample in PBS was added to 180 μL of either PBS (iso-osmotic) or sterile distilled water (hypo-osmotic) in a 96 well PCR plate, and incubated at room temperature for either 30 s or five min. After incubation in iso-osmotic (PBS) or hypo-osmotic (water) conditions, samples were heated at 58 °C for five min. The heat resistance levels of isolates were measured by the modified MPN method described above. In some cases, cells were transferred back from water into PBS buffer (for 15 min) just prior to heat treatment (58 °C for four min), and in order to assess the reversibility of this osmotic effect on heat resistance.

### 2.4 Calculation of D58 curves and Weibull parameters

D-values (the time required to inactivate 90% of the microbial population at a target temperature) were calculated for each isolate based on plotting survival curves (i.e., log10 MPN/mL across time (min) at 58 °C) and deriving a best-fit second-order polynomial curve (Excel, Microsoft). The Weibull model was also applied to describe bacterial resistance to heat by using the GInaFiT tool version 1.8 (KU Leuven, Leuven, Belgium). This software, as detailed by Geeraerd et al. (2005), offers several parameters: *δ*, denoting the time taken for the first decimal reduction of the microbial population; p, representing the shape of an inactivation curve (*p < 1* for upward concavity, and *p > 1* for downward concavity. *p = 1* for a linear survival curve); and 4D reduction, indicating the time required to inactivate 99.99% of the microbial population. For both the second-order polynomial curve and the Weibull model equations, a minimum of four data points were employed. Parameters are presented as the mean ± standard deviation, based on at least three independent experiments. All correlation coefficients (R^2^) were > 0.94.

### 2.5 Statistical analysis of data

After performing each experiment in triplicate (i.e., performed independently on three separate days), data were presented as means ± the standard deviations of the mean and statistically analyzed using one- or two-way analysis of variance (ANOVA) in GraphPad Prism (version 9.5.1; GraphPad Software Inc.). The significance of differences was determined using the Bonferroni multiple comparisons test, with a significance level set at *p* < 0.05. For the rapid induction of a heat-resistant phenotype in wastewater ExPEC strains, an unpaired Student’s t-test was performed using R software version 4.4.0, with a *p*-value < 0.05 as statistically significant.

## 3 Results

**Figure 1:**
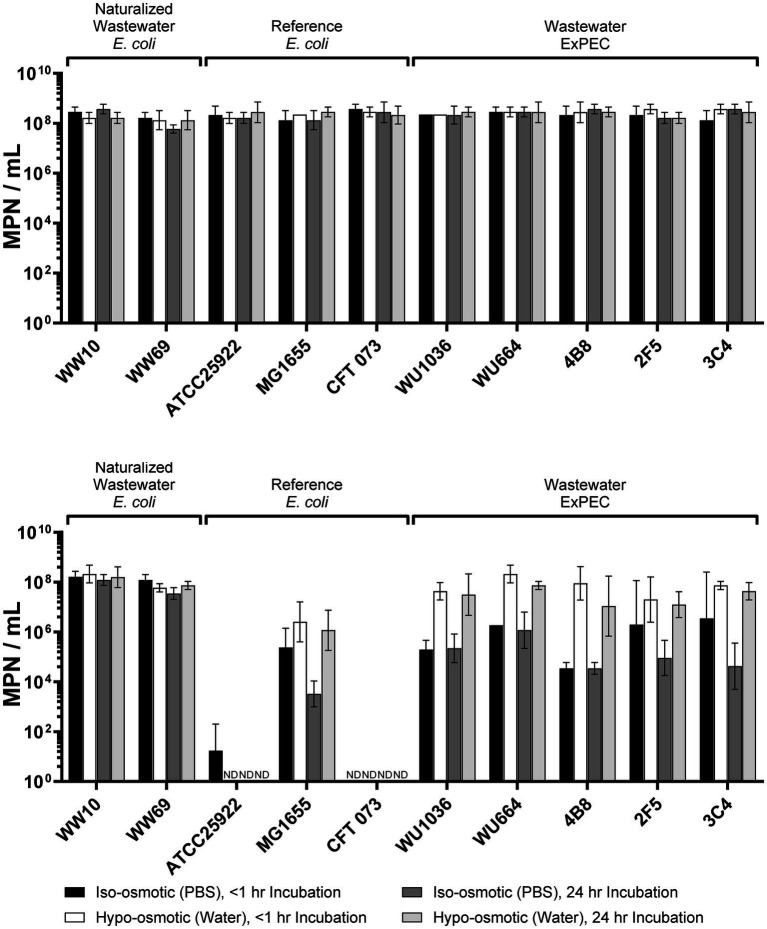
*Top panel* - Survivability of *Escherichia coli* strains after 1 or 24 h of exposure to cold stress (room temperature) and nutrient depletion, as well as different osmotic conditions [hypo-osmotic vs. iso-osmotic (shaded bars)]. Overnight bacterial cultures were washed 2 times in PBS at room temperature (cold stress) and resuspended in sterile distilled water (nutrient deprivation/hypo-osmotic stress) or PBS (nutrient deprivation/iso-osmotic stress) for 1 h or 24 h and survivability assessed by culture. Note that laboratory conditions experienced by bacteria prior to heat treatment, did not affect culturability of any of the strains over these time periods. *Bottom panel* - Survivability of pre-conditioned *Escherichia coli* strains (as in top panel) after heat stress at 58 °C for 5 min. Error bars in both panels indicate the standard deviation across three independent replicate experiments performed. ND, not detected. See Supplementary Tables S1, S2 for statistical summary of data.

Invariably, laboratory manipulations impose stressful conditions on bacteria, and for this reason it was important to first assess what impact our experimental culture conditions had on the viability of *E. coli* strains before heat treatment. In our experiments, all strains were exposed to cold stress (e.g., setting up experiments at room temperature) and nutrient depletion (PBS or sterile distilled water) before heat treatment, albeit the latter condition also imposed osmotic stress on cells as well. Exposure of strains to cold stress (room temperature), nutrient deprivation (incubation in PBS/water) and osmotic shock (water versus PBS) for 1 or 24 h had no significant effect on the culturability of any of the *E. coli* strains in our study (Figure 1, top panel; see Supplementary Table S1 for results of statistical analysis), suggesting that all strains used in our experiments were tolerant of these stressors for up to 24 h of incubation and with no effect on their culturability. As such, any reduction in culturability observed in cell populations after heat-treatment could be attributed to application of heat and not directly due to other stressors - albeit additive/synergistic effects could not be ruled out.

The application of heat stress (58 °C for 5 min) to strains led to significant differences in culturability among all *E. coli* strains (Figure 1, bottom panel; see Supplementary Table S1 for results of statistical analysis). At one end of the spectrum, the clinical reference ExPEC strain, CFT073, was completely inactivated upon heat treatment at 58 °C regardless of pre-treatment conditions (>8 log10 decline in culturability; Figure 1, bottom panel) and for this reason it was deemed to be the most heat-susceptible of all *E. coli* strains tested. At the other end of the spectrum were the naturalized wastewater *E. coli* (WW10, WW69) which were completely resistant to heat treatment, and for which there was no significant loss in culturability of cells after heat treatment regardless of pre-treatment conditions (Figure 1; Supplementary Table S1). For the other control strains, significant declines in ATCC25922 were observed (>6 log10 MPN/mL) under all pre-treatment conditions, and this strain was deemed to have a heat-susceptible phenotype like CFT073 (Figure 1; Supplementary Table S1). By contrast, the laboratory control strain MG1655 displayed an intermediate level of resistance to heat, and interestingly, cells pre-conditioned under hypo-osmotic conditions (water) for 24 h were significantly more resistant to heat than cells pre-conditioned under iso-osmotic (PBS) conditions for 24 h, suggesting that exposure to water may be a trigger of a heat-resistance phenotype in this strain (Figure 1; Supplementary Tables S1, S2). Indeed, under hypo-osmotic pre-treatment conditions, the culturability of MG1655 cells declined by only ~2 log10 MPN/mL, whereas cells pre-treated under iso-tonic conditions (PBS) for 24 h declined by ~4 log10 MPN/mL.

In terms of ExPEC, no significant loss in culturability was observed in any of the strains after heat treatment, provided that the cells were first pre-incubated in sterile distilled water before heat treatment (Figure 1; Supplementary Table S1). As such, the W-ExPEC displayed an extreme heat resistance phenotype like naturalized *E. coli* under these conditions; however, unlike the naturalized strains, pre-treatment of ExPEC under iso-osmotic conditions (i.e., PBS) resulted in significantly less resistance to heat, with cell cultures declining by ~2–4 log10 MPN/mL (Figure 1; Supplementary Table S2). This hypo-osmotic induction of a heat-resistant phenotype was most notable in strains 4B8 and 3C4 (both presumptive BBEC strains), where incubation of cells in sterile distilled water for <1 or 24 h for each strain, respectively, led to a 1,000-fold increase in resistance to heat in both strains compared to cells incubated in PBS for these same time periods (Figure 1; Supplementary Table S2). A significant increase in heat resistance under hypo-osmotic conditions was observed for all of the W-ExPEC strains when comparing the 24 h pre-treatment condition to PBS or water, except for 2F5, suggesting that exposure to osmotic stress (i.e., water) acts as an anticipatory trigger of the induction of an extreme heat resistant phenotype in most wastewater treatment-resistant ExPEC strains.

**Figure 2:**
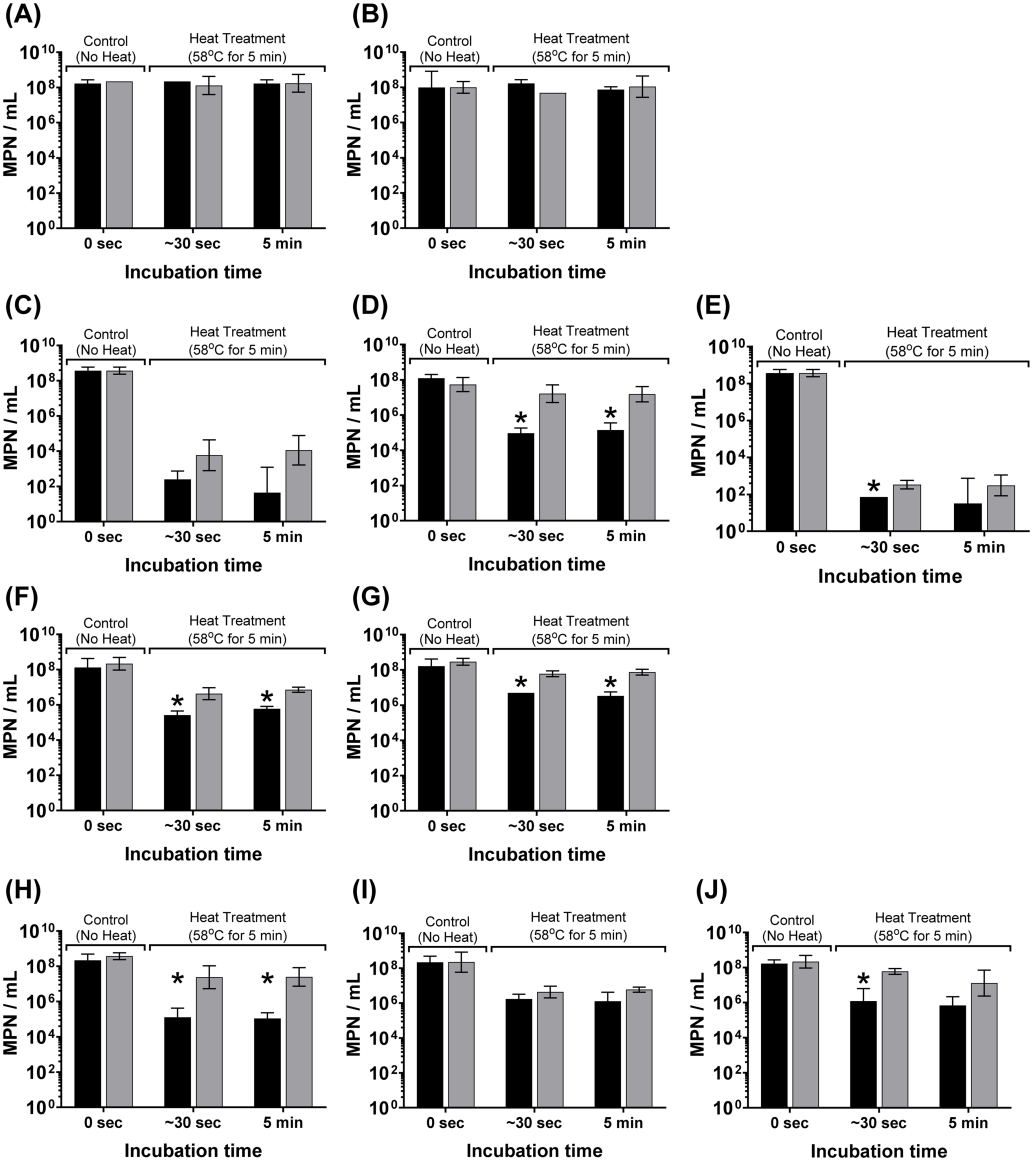
Rapid induction of a heat-resistant phenotype in wastewater ExPEC strains due to hypo-osmotic stress. Bacterial cells were grown overnight, washed twice in PBS, and re-suspended in sterile distilled water [hypo-osmotic (grey bars)] or PBS [iso-osmotic (black bars)] for 30 s or 5 min before heat treatment at 58 °C for 5 min. Error bars indicate the standard deviation among three completely independent replicate trials. Asterix indicates significance (*p* < 0.05) between hypo-osmotic and iso-osmotic conditions for that designated time point based on an unpaired Student’s t-test. Panels correspond to the following bacterial strains: (**A**) WW10, (**B**) WW69, (**C**) ATCC25922, (**D**) MG1655, (**E**) CFT073, (**F**) WU1036, (**G**) WU664, (**H**) 4B8, (**I**) 2F5, and (**J**) 3C4.

The osmotic induction of this extreme heat resistant phenotype in W-ExPEC occurred rapidly, within seconds of exposure of bacteria to sterile distilled water (Figure 2). For most ExPEC strains (except 2F5), incubation of cells in sterile distilled water for as little as ~30 s led to a statistically significant 10- to >100-fold increase in resistance to heat (Figure 2). The effect was most notable for 4B8 and the control strain MG1655 where heat resistance increased ~188 fold and 177-fold, respectively, after only 30 s of exposure to water compared to cells incubated in PBS (Figure 2). Notably, this effect was also reversible in that when cells were incubated in water and then returned to PBS, they once again adopted a more susceptible phenotype to heat (Supplementary Figure S1).

**Figure 3:**
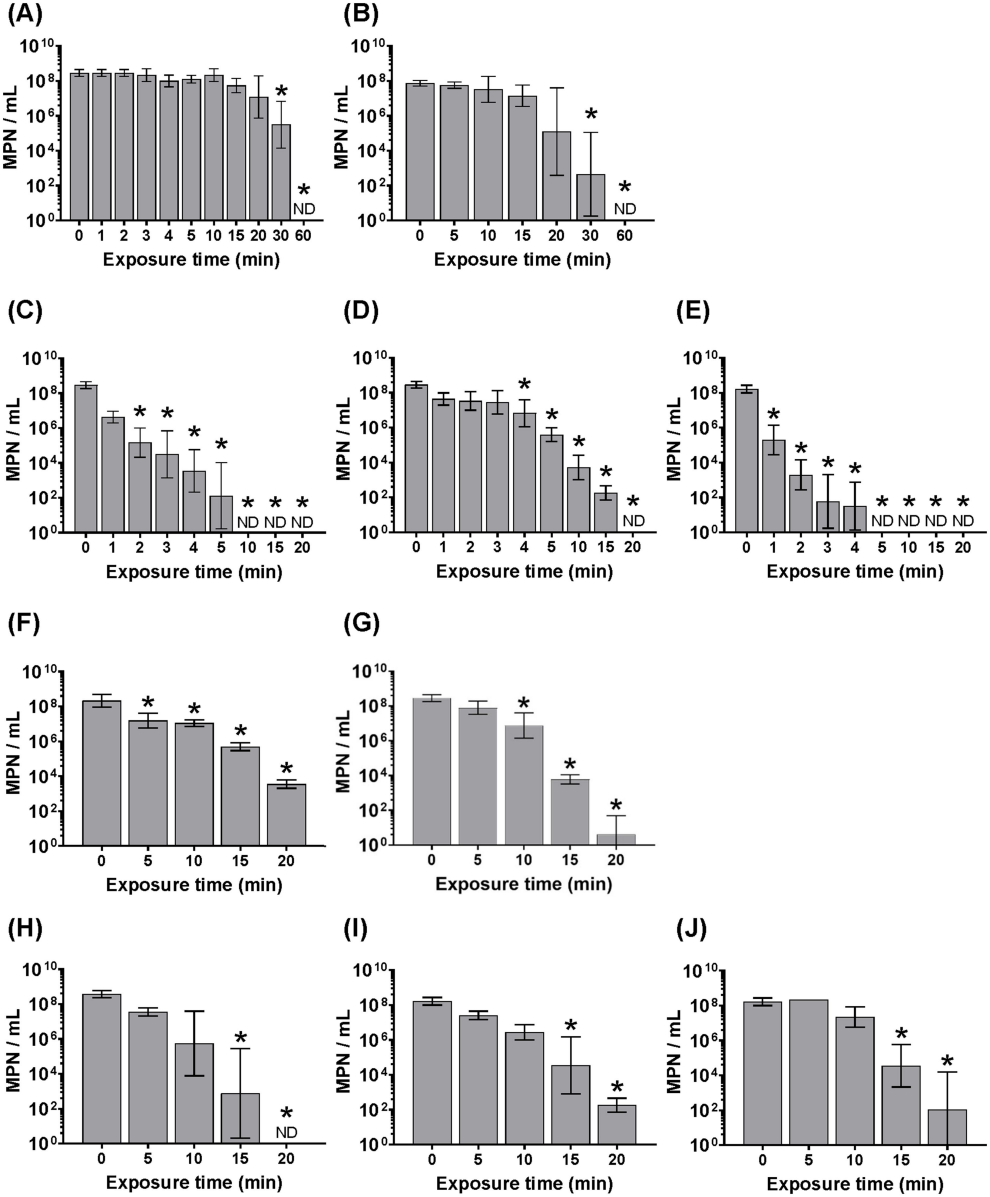
Assessing heat resistance of hypo-osmotic pre-conditioned (~1 h) *Escherichia coli* strains based on varying the length of exposure to 58 °C. All strains were cultured overnight, washed 2 times in PBS, and resuspended in sterile distilled water before heat treatment. Error bars indicate the standard deviation among three independent experiments performed. ND, not detected. Asterix indicates significance (*p* < 0.05) between the designated time point and the zero- time point using one-way ANOVA and the Bonferroni multiple comparisons test. Panels correspond to the following bacterial strains: (**A**) WW10, (**B**) WW69, (**C**) ATCC25922, (**D**) MG1655, (**E**) CFT073, (**F**) WU1036, (**G**) WU664, (**H**) 4B8, (**I**) 2F5, and (**J**) 3C4.

Most W-ExPEC strains that were hypo-osmotically pre-conditioned were still culturable after 20 min at 58 °C (the exception being 4B8 that survived 15 min), whereas the clinical ExPEC strain CFT073 was reduced to non-culturable levels after only 5 min (i.e., >8 log10 MPN/mL), and ATCC 25922 after 10 min (Figure 3). By contrast the naturalized wastewater *E. coli* strains were reduced to a non-culturable state after 60 min of exposure to 58 °C (i.e., WW10), suggesting that naturalized strains were actually more resistant to heat overall when compared to all other strains, including W-ExPEC (Figure 3). The D58 values (i.e., time required for 1 log10 inactivation at 58 °C) confirmed that naturalized wastewater *E. coli* strains were the most heat-resistant followed by the W-ExPEC strains, then MG1655, ATCC25922 and lastly, CFT073 (Table 2). Complementary to this analysis, the delta and the 4D reduction values provided by the Weibull model, indicating the time necessary to achieve 90 and 99.99% inactivation at 58 °C respectively, consistently supported these findings. Notably, the time required for the W-ExPEC strains to reach a 4 log10 reduction surpassed 10 min, showing a statistically-significant deviation in their thermal tolerance when compared to the reference clinical ExPEC strain, CFT073 (Table 2; Supplementary Table S3).

**Table 2:** Thermal kinetic parameters (mean ± standard deviation) of second-order polynomial regression and weibull models for wastewater ExPEC survival at 58 °C.

	2nd Polynomial Regression	Weibull model		
*Escherichia coli* Strains	D-value (min)^a^	δ (min)^a^	*p* ^a^	4D Reduction (min)^a^
WW10	20.662 ± 5.338	17.816 ± 9.747	1.871 ± 0.889	38.4 ± 7.373
WW69	13.132 ± 0.636	10.798 ± 5.107	1.456 ± 0.386	28.9 ± 9.45
ATCC 25922	0.782 ± 0.5	0.691 ± 0.299	0.792 ± 0.122	3.933 ± 0.586
MG1655	3.554 ± 2.113	3.457 ± 1.318	1.365 ± 0.23	9.767 ± 3.134
CFT073	0.325 ± 0.118	0.176 ± 0.156	0.617 ± 0.185	1.483 ± 0.52
WU1036	7.966 ± 2.5	7.617 ± 1.387	1.714 ± 0.046	17.2 ± 3.175
WU664	6.077 ± 1.8	6.807 ± 1.244	1.857 ± 0.399	14.667 ± 0.808
4B8	6.025 ± 2.244	6.769 ± 3.108	2.174 ± 0.783	12.533 ± 3.775
2F5	7.036 ± 2.613	8.037 ± 3.694	2.045 ± 0.789	16.133 ± 2.996
3C4	10.004 ± 2.203	8.764 ± 1.905	2.205 ± 0.189	16.467 ± 3.126

^a^Values represent the mean from three independent replicate trials ± standard deviation.

**Figure 4:**
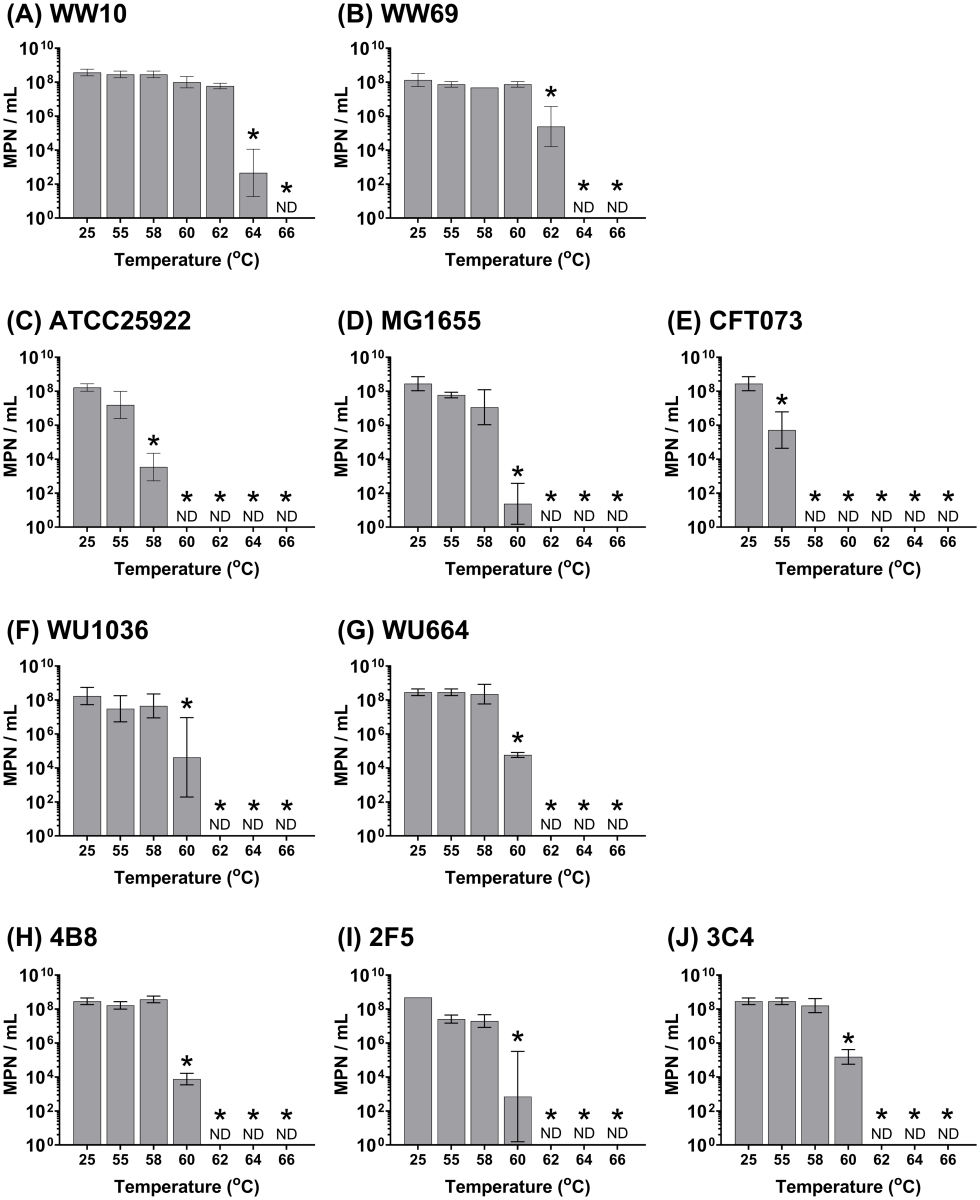
Assessing heat resistance of hypo-osmotic preconditioned *Escherichia coli* strains based on exposure to varying temperatures for 5 min. All strains were cultured overnight, washed 2 times in PBS, and resuspended in sterile distilled water before heat treatment. Error bars indicate the standard deviation among three independent experiments performed. ND, not detected. Asterix indicates significance between the designated temperature and the 25 °C temperature point using two-way ANOVA and the Bonferroni multiple comparisons test. Panels correspond to the following bacterial strains: (**A**) WW10, (**B**) WW69, (**C**) ATCC25922, (**D**) MG1655, (**E**) CFT073, (**F**) WU1036, (**G**) WU664, (**H**) 4B8, (**I**) 2F5, and (**J**) 3C4.

For all W-ExPEC strains, the upper thermal tolerance level (i.e., the temperature at which an exposure for 5 min resulted in the complete absence of culturable cells) was 62 °C, whereas naturalized wastewater strains had upper thermal tolerance levels of 64 °C and 66 °C for WW69 and WW10 strains, respectively (Figure 4). In contrast, the upper thermotolerance of CFT073 was only 58 °C, 60 °C for ATCC25922, and 62 °C for MG1655 (Figure 4).

## 4 Discussion

Sewage treatment processes have been shown to select for resistant microbes, including *E. coli* belonging to the ExPEC pathotype (Anastasi et al., 2010; Anastasi et al., 2013; Calhau et al., 2015; Adefisoye and Okoh, 2016; Zhi et al., 2020; Yu et al., 2022a), but to date, no studies have addressed the phenotypic characteristics of stress resistance in these wastewater-derived ExPEC strains. Herein we demonstrate that wastewater derived ExPEC display a robust heat-resistant phenotype.

W-ExPEC isolated from chlorinated sewage and treated wastewater could withstand 58 °C for 5 min with no loss in culturability - a phenotype similar to the tLST-mediated resistance in naturalized wastewater *E. coli* (Wang et al., 2020). By comparison, the ATCC reference ExPEC strain, CFT073, became non-culturable after only 5 min (i.e., >8 log10 decline in culturability). Interestingly, this extreme heat-resistance phenotype observed in W-ExPEC was dependent on pre-exposure of cells to hypo-osmotic conditions and W-ExPEC strains were 10–1,000 times more resistant to heat when pre-conditioned in water compared to cells pre-conditioned in iso-osmotic PBS. This effect is counter-intuitive since hyper-osmotic shock, but not hypo-osmotic shock, is known to increase heat resistance of *E. coli*. In brief, the deformation of membranes after osmotic upshock activates transport proteins within a few seconds, increasing the cytoplasmic concentration of solutes including potassium, glutamate and glycine-betaine (Larsen et al., 1987; Roeßler and Müller, 2001; Poolman et al., 2002). The high concentration of solutes protects macromolecules against denaturation by heat on the basis of a thermodynamical principle termed ‘preferential hydration’ or ‘preferential exclusion’ (Parsegian et al., 2000; Ruan et al., 2003; Pleitner et al., 2012). Conversely, hypo-osmotic shock results in the release of osmolytes through mechano-sensitive channels (Buda et al., 2016); a response that also induces biophysical changes to the cytoplasmic membrane in a matter of seconds. The extremely rapid and reversible induction of the heat-resistant phenotype under hypo-osmotic conditions indicates that this response is also related to solute transport rather than *de novo* protein synthesis. This mechanism is in contrast to stressors such as starvation that induces heat resistance in *E. coli* but for which adaptation is observed on a scale of hours rather than seconds and is dependent on *de novo* protein synthesis (Yu et al., 2022b). Previous studies suggest that changes in fluidity and permeability of the cell envelope contribute to bacterial thermotolerance. Shigapova et al. (2005) demonstrated that transient membrane fluidization and permeabilization induced by sublethal heat stress and benzyl alcohol facilitates survival under heat stress. Similarly, Guyot et al. (2010) showed that *E. coli* heat resistance is associated with modifications in membrane structure that increase its fluidity and permeability. Hypo-osmotic stress enhances the permeability of both outer and inner membranes without compromising cell viability (Kumar et al., 2023), and collectively these studies imply that envelope remodeling may play a critical role in stress adaptation. To our knowledge there is no information on plausible mechanisms on how the expulsion of solutes or changes in cell envelope under hypo-osmotic shock may increase heat resistance of *E. coli*. Nevertheless, our findings provide some tantalizing evidence to suggest that exposure to a hypo-osmotic water environment may drive W-ExPEC into a resistant state and that may be important for their survival against sewage/wastewater treatment – a phenomenon observed by several research groups, including our own (Anastasi et al., 2010; Anastasi et al., 2013; Calhau et al., 2015; Adefisoye and Okoh, 2016; Zhi et al., 2020; Yu et al., 2022a).

The data also raise the specter that pathogenic strains of *E. coli* may be evolving resistance to wastewater treatment. In terms of ExPEC, Yu et al. (2022a) recently demonstrated a relatively high degree of relatedness between ExPEC and naturalized wastewater *E. coli* at the accessory genome level, suggesting that these strains may share a common backbone of genes important for promoting their survival in wastewater ecosystems. Interestingly, although naturalized wastewater *E. coli* are believed to be non-pathogenic (Yu et al., 2022b) these strains possess upwards of 36 different ExPEC-related virulence genes (Zhi et al., 2016), suggesting that these virulence genes may be important for both naturalized *E. coli* and W-ExPEC to survive within the wastewater environment (and possibly in an aquatic ecosystem). These genes include iron-acquisition genes such as the ferrienterobactin multi-enzyme sythetase complex, the enterobactin-related receptor transport pathways, and the yersiniabactin siderophore biosynthetic pathway and receptor/transport system - genes which are important in the survival of ExPEC in the osmotically- and iron-stressed environment of the urinary tract (Mann et al., 2017; Terlizzi et al., 2017), but which may also be important in iron acquisition in wastewater. Naturalized wastewater strains also possessed the ExPEC-associated virulence gene *irp* – a gene associated with protection against protozoan grazing and predation (Adiba et al., 2010), and which is particularly interesting since microbial predation represents an important wastewater treatment process. W-ExPEC and naturalized wastewater *E. coli* also appear to share a common backbone of antibiotic resistance genes (Yu et al., 2022a). Consequently, this sharing of ExPEC-related virulence genes and a common set of antibiotic resistance genes among naturalized wastewater *E. coli* and W-ExPEC suggests that these genes may be required for these strains to survive in a wastewater/sewage environment.

While the exact evolutionary relationship between naturalized wastewater *E. coli* and W-ExPEC is uncertain, their accessory genome structure seems to imply the potential for horizontal gene transfer of treatment resistance, albeit there is no concrete evidence of this phenomenon to date. Reflecting this, the tLST is present in naturalized wastewater *E. coli*, but lacking in W-ExPEC even though the tLST has been shown to be an important mechanism of heat, chlorine and oxidant resistance (Wang et al., 2020) and is located on a transposable element. Furthermore, hypo-osmotic induction of a heat-resistant phenotype in W-ExPEC, but not naturalized strains, suggests that the evolutionary trajectory toward water treatment resistance may not be linear, and supports the idea that bacteria have an evolutionary toolbox of mechanisms to develop resistance to treatment and disinfection.

On the surface, the evolution of heat resistance in both naturalized wastewater *E. coli* and W-ExPEC appears paradoxical, since wastewater within a treatment plant rarely exceeds 25 °C. However, the management of municipal sewage entails the treatment of both the solids (i.e., biosolids/sludge) and the liquids phase (i.e., wastewater) of municipal wastes, and heat is often used for treatment of the biosolids/sludge. For example, according to U.S. EPA regulations [40 CFR Part 503] (USEPA, 1993), Class A human biosolids requires treatment at temperatures of 55 °C for 24 h to meet adequate sanitization requirements, and Class A biosolids can be used as soil amendments for agricultural crops (or gardening soil). For Class B biosolids, composting temperatures of 40 °C or higher are needed, and must be maintained for five days, but for which for four hours during the five-day period the compost pile should exceed 55 °C. Although the duration of exposure for composting is significantly longer than the times explored in this paper (i.e., 55 °C for 4 h for Class B biosolids compared to 58 °C for up to 60 min in this paper), the composting temperatures are also lower (55 °C compared to 58 °C). It is possible that induction of this heat-resistant phenotype may be important for these pathogens to survive biosolids composting and represent a research area we are currently exploring. Interestingly, the gradual acclimation of *E. coli* to high incubation temperatures (i.e., 41.5 °C) has been shown to result in inducible thermotolerance at typically lethal temperatures (i.e., 50 °C). Reflecting this, Isobaev et al. (2014) used a programmable incubator that simulated composting conditions (i.e., gradual increase in heat from room temperature to 62 °C), and demonstrated that a strain of *E. coli* originally isolated from compost, could survive >2.5 days at >55 °C in the laboratory, and with temperatures peaking at 62 °C, demonstrating the incredible range of thermotolerance in *E. coli* as a species. The work presented in this paper provides a basis for addressing heat-resistance in W-ExPEC and their potential ability to survive at temperatures and conditions relevant to biosolids/sludge waste treatment.

Our findings also have important implications for the water/wastewater industry in terms of understanding treatment efficacy. Firstly, most bench-scale inactivation studies using chlorine, UV, and/or ozone use PBS as a diluent for bacteria (Gomes et al., 2016; Ding et al., 2019; Szeto et al., 2020). Our data suggest that PBS increases susceptibility of cells to oxidizing heat, and contrasts starkly with the rapid induction of a resistant phenotype when W-ExPEC are exposed to water only. Exposure to hypo-osmotic conditions more closely mimics the natural environment that ExPEC experience when transiting from a human host to a wastewater treatment plant, and our data suggest that a hypo-osmotic state may be an anticipatory trigger of resistance for these cells. Secondly, most disinfection studies typically employ laboratory strains of *E. coli* as surrogates for understanding treatment efficacy (Mazzola et al., 2006; Martinelli et al., 2017; Pihen et al., 2023). Our data, and that of others (Gadzella and Ingham, 1994; Dlusskaya et al., 2011), clearly demonstrates that laboratory strains of *E. coli* are not particularly useful surrogates for understanding the overall levels of heat-resistance in *E. coli* as a species. We contend that future studies in water disinfection should focus on understanding the kinetics of inactivation of naturalized wastewater *E. coli* or W-ExPEC, as opposed to laboratory strains, in order to better understand waterborne disease risks associated with stress-resistant bacteria.

From a public health perspective, a very important question relates to understanding how W-ExPEC might be evolving resistance to wastewater treatment. The evolution of wastewater-treatment resistance for a pathogen such as ExPEC requires that the microbe maintain its pathogenic properties in a human/animal host while also undergoing natural selection in the wastewater environment. In order for this to happen, sufficient microbial traffic must cycle between these divergent environments for both treatment-resistance and pathogenesis to become fixed in a population. So, what evidence is there for the cyclic trafficking of ExPEC between the host environment (humans) and non-host environment (e.g., wastewater/sewage)?

It is estimated that greater than 10 million physician visits occur each year in the U.S. alone due to urinary tract infections and for which the vast majority of these infections are caused by ExPEC (Xie et al., 2006; Flores-Mireles et al., 2015; Pitout and DeVinney, 2017; Russell et al., 2018). This is likely a gross underestimation of true prevalence given that many people may not seek medical attention when infected. Paulshus et al. (2019), observed that up to 10% of *E. coli* in untreated wastewater was considered extended spectrum beta-lactamase (ESBL) producing ExPEC, leading the authors to speculate that ExPEC may represent either a resident population within wastewater systems, or that ExPEC infections are underreported in the community. Importantly, research demonstrates that ExPEC typically establish infection by first asymptomatically colonizing the gastrointestinal tract of a human/animal host, subsequently invading the gut lining and disseminating to the urinary tract [UPEC], bloodstream [BBEC] or meninges [NMEC] (Bower et al., 2005; Russell et al., 2017). In terms of NMEC, neonates acquire the bacteria during the birthing process from their infected mothers as asymptomatic carriers of these strains (Camacho-Gonzalez et al., 2013). The fact that certain ExPEC may be part of the gut microbiome explains why these strains may be so dominant in wastewater.

There is also evidence demonstrating that the strains surviving wastewater treatment typically represent sequence types associated with human infection (Zhi et al., 2020; Yu et al., 2022a). For example, in a single sample of chlorinated sewage, Zhi et al. (2020) isolated 13 different W-ExPEC isolates comprising 6 different sequence types (ST131, ST80, ST625, ST127, ST95 and an unknown ST), and for which 11 of the isolates belonged to 4 sequence types commonly associated with human ExPEC infections [ST131, ST80, ST127, and ST95]. In a survey of wastewater treatment plants in the U.S., Hoelle et al. (2019) also observed a relative abundance of ESBL-ExPEC strains associated with human pathogenic ST131 and ST648 lineages, findings that are in alignment with those of Zhi et al. (2020) and Yu et al. (2022a). As noted previously, Anastasi et al. (2010, 2013) observed that ExPEC strains were more prevalent at different stages of sewage/wastewater treatment, including after chlorination and UV disinfection. From these studies it is clear that human-infectious ExPEC undergo natural selection, leading to their differential survival across the wastewater treatment environment.

How, then, could these treatment resistant strains circulate back into the human population? There is growing speculation that water may be an important, and underestimated, vehicle of transmission for ExPEC (Graham et al., 2021). For example, an epidemiological study linked swimming in a natural water body to increased risk of urinary tract infections (UTIs) largely caused by ExPEC (Søraas et al., 2013). Based on virulence gene analysis, Ebomah et al. (2018) observed that 44.9% of *E. coli* isolates from treated wastewater effluents that impacted a recreational beach were deemed potential ExPEC. The United States Environmental Protection Agency (U.S. EPA) estimates that as many as 40,000 sewer overflows occur each year, and up to 500,000 km of coastlines, rivers and streams do not meet ambient microbial water quality guidelines for recreation as a result of human and animal waste contamination (USEPA, 2007a, 2007b). In a study by Tanner et al. (2019), extended spectrum beta-lactamase resistant (ESBL) bacteria were found in 6.4% of drinking water samples that failed bacteriological water quality parameters in the U.S. (i.e., total coliforms), for which several of the ESBLs identified were *E. coli*, and which led the authors to conclude that drinking water may be an underestimated vehicle for transmission of ExPEC into the community. It is estimated that 50% of drinking water treatment plants in the U.S. are impacted by wastewater effluents, and in some cases wastewater effluents account for >50% (and as high as 90%) of river flow volumes in the U.S. (Rice and Westerhoff, 2015, 2017). ESBL *E. coli* have been routinely found in surface water samples and irrigation water samples, and some of these studies report ExPEC as dominant strains comprising these ESBL populations (Franz et al., 2015; Njage and Buys, 2015; Müller et al., 2016; Gomi et al., 2017; Gekenidis et al., 2018). Also noted above, human biosolids are widely used as soil amendments in agriculture and gardening. Based on virulence gene characterization, Hossain et al. (2021) observed that as many as 51% of *E. coli* strains isolated from faecal sludge carried at least one ExPEC related gene, and 4% carried at least four ExPEC genes, and all of the latter were multidrug resistant. As far as we are aware, no studies have looked at prevalence of ExPEC in biosolids used as agricultural/gardening amendments. Collectively, there is considerable indirect evidence to support the idea that wastewater treatment resistant ExPEC have a multitude of ways of potentially trafficking back into the human population through the food-water nexus, thereby maintaining their pathogenesis.

The potential emergence of treatment-resistant pathogenic bacterial populations in wastewater is worrying, especially considering that *E. coli* is only one species in a vast wastewater microbiome. Indeed, the very same natural selection forces potentially driving treatment resistance in ExPEC could be driving the evolution of other pathogenic microbial populations (e.g., coliforms) in wastewater. We believe it is imperative that more research be done to understand the fundamental mechanisms of phenotypic treatment resistance in these strains in order to better assess the risks that these pathogens pose to human health from exposure to water contaminated with sewage or wastewater.

## Data Availability

The raw data supporting the conclusions of this article will be made available by the authors, without undue reservation.
