# Supplementary material for: Hypo-osmotic stress is an anticipatory trigger of heat-resistance in presumptive extraintestinal pathogenic Escherichia coli isolated from treated sewage
Source: Front Microbiol. 2025 Oct 8;16:1676613. doi: 10.3389/fmicb.2025.1676613 (PMC12540446; doi:10.3389/fmicb.2025.1676613)
Supplement: Supplementary file 1 [file Data_Sheet_1.docx]

**(A) WW10**

**(B) WW69**

**(C) ATCC25922**

**(D) MG1655**

**(E) CFT073**

**(F) WU1036**

**(G) WU664**

**(H) 4B8**

**(I) 2F5**

**(J) 3C4**

**Supplemental Figure S1.** Reversibility of osmotic triggers of heat resistance in wastewater ExPEC strains pre-conditioned in water. Overnight bacterial cultures were also washed twice in sterile distilled water and resuspended in PBS (iso-osmotic [black bars]) or sterile distilled water (hypo-osmotic [grey bars]) for 30 sec or 5 min before heat treatment at 58^o^C for 5 min. Error bars indicate the standard deviation among three completely independent replicate trials. * indicates significance between hypo-osmotic and iso-osmotic conditions for that designated time point based on an unpaired Student’s t-test.
