# Supplementary material for: Hypo-osmotic stress is an anticipatory trigger of heat-resistance in presumptive extraintestinal pathogenic Escherichia coli isolated from treated sewage
Source: Front Microbiol. 2025 Oct 8;16:1676613. doi: 10.3389/fmicb.2025.1676613 (PMC12540446; doi:10.3389/fmicb.2025.1676613)
Supplement: Supplementary file 2 [file Table_1.docx]

**Supplemental Table S1**. Two-way ANOVA summary statistics table evaluating the effect of pre-experimental conditions (i.e., PBS [iso-osmotic] or sterile distilled water [hypo-osmotic]) on the survivability of *E. coli* strains before and after application of heat stress (58^o^C for 5 min). Significant *p* values shaded in grey [<0.05].

|  |  | No Heat | | | | | Effect of heat stress on the survivability of bacterial cells incubated under similar pre-experimental conditions (water [hypo-osmotic] or PBS [iso-osmotic]) | | | | |
| --- | --- | --- | --- | --- | --- | --- | --- | --- | --- | --- | --- |
| *Escherichia coli* Strains | | <1 hr water  vs.  <1 hr PBS (pre-condition control) ^a^ | 24 hrs water  vs.  <1 hrs PBS (pre-condition control) ^a^ | <1 hr water  vs.  24 hrs water | <1 hr PBS  Vs  24 hrs PBS | <1 hr water (no heat)  vs.  <1 hr water (heat) | | 24 hrs water (no heat)  vs.  24 hrs water (heat) | <1 hr PBS (no heat)  vs.  < 1 hr PBS  (heat) | 24 hr PBS (no heat)  vs  24 hrs PBS  (heat) |  |
| Control Strains | ATCC 25922 | ns ^b^ | ns | ns | ns | <0.001 | | <0.001 | <0.001 | <0.001 |  |
|  | MG1655 | ns | ns | ns | ns | 0.02 | | 0.002 | <0.001 | <0.001 |  |
|  | CFT073 (Clinical ExPEC) | ns | ns | ns | ns | <0.001 | | <0.001 | <0.001 | <0.001 |  |
| Naturalized Wastewater *E. coli* Strains | WW10 | ns | ns | ns | ns | ns | | ns | ns | ns |  |
|  | WW69 | ns | ns | ns | ns | ns | | ns | ns | ns |  |
| Wastewater ExPEC  Strains | WU1036 | ns | ns | ns | ns | ns | | ns | <0.001 | <0.001 |  |
|  | WU664 | ns | ns | ns | ns | ns | | ns | <0.001 | <0.001 |  |
|  | 4B8 | ns | ns | ns | ns | ns | | ns | <0.001 | <0.001 |  |
|  | 2F5 | ns | ns | ns | ns | ns | | ns | ns | 0.003 |  |
|  | 3C4 | ns | ns | ns | ns | ns | | ns | ns | <0.001 |  |

^a^ Assessment of the survivability of bacterial cells in water (hypo-osmotic state) compared to controls (<1 hr PBS [iso-osmotic]).

^b^ ns – Not Significant (p value > 0.05)
