# Supplementary material for: Hypo-osmotic stress is an anticipatory trigger of heat-resistance in presumptive extraintestinal pathogenic Escherichia coli isolated from treated sewage
Source: Front Microbiol. 2025 Oct 8;16:1676613. doi: 10.3389/fmicb.2025.1676613 (PMC12540446; doi:10.3389/fmicb.2025.1676613)
Supplement: Supplementary file 3 [file Table_2.docx]

**Supplemental Table S2**. Two-way ANOVA summary statistics table evaluating the effect of osmotic shock on *E. coli* survival against heat stress (58^o^C for 5 min). Significant *p* values shaded in grey [<0.05].

|  |  | Effect of Pre-experimental Culture Conditions on Survivability After Heat Treatment | | |
| --- | --- | --- | --- | --- |
| *Escherichia coli* Strains | | <1 hr water (heat)  vs.  <1 hr PBS (heat) | 24 hr water (heat)  vs.  24 hrs PBS  (heat) | <1 hr water (heat)  vs.  24 hrs PBS  (heat) |
| Control Strains | ATCC 25922 | ns ^a^ | ns | ns |
|  | MG1655 | ns | <0.001 | <0.001 |
|  | CFT073 (Clinical ExPEC) | ns | ns | ns |
| Naturalized Wastewater *E. coli* Strains | WW10 | ns | ns | ns |
|  | WW69 | ns | ns | ns |
| Wastewater ExPEC Strains | WU1036 | <0.001 | <0.001 | <0.001 |
|  | WU664 | <0.001 | <0.001 | <0.001 |
|  | 4B8 | <0.001 | 0.001 | <0.001 |
|  | 2F5 | ns | ns | ns |
|  | 3C4 | ns | 0.006 | 0.003 |

^a^ ns – Not Significant (*p* >0.05)
