# Supplementary material for: Hypo-osmotic stress is an anticipatory trigger of heat-resistance in presumptive extraintestinal pathogenic Escherichia coli isolated from treated sewage
Source: Front Microbiol. 2025 Oct 8;16:1676613. doi: 10.3389/fmicb.2025.1676613 (PMC12540446; doi:10.3389/fmicb.2025.1676613)
Supplement: Supplementary file 4 [file Table_3.docx]

**Supplemental Table S3**. Two-way ANOVA summary statistics table evaluating the significant variance in 4D reduction values of *E. coli* strains at 58^o^C. Significant *p* values shaded in grey [<0.05].

|  | |  | 4D Reduction | | | | | | | | | |
| --- | --- | --- | --- | --- | --- | --- | --- | --- | --- | --- | --- | --- |
|  | | | Control Strains | | | Naturalized Wastewater *E. coli* Strains | | Wastewater ExPEC Strains | | | | |
| *Escherichia coli* Strains | | | ATCC25922 | MG1655 | CFT073 (Clinical ExPEC) | WW10 | WW69 | WU1036 | WU664 | 4B8 | 2F5 | 3C4 |
| Control Strains | ATCC25922 | | - | ns ^a^ | ns | <0.001 | <0.001 | 0.002 | 0.03 | ns | 0.006 | 0.004 |
|  | MG1655 | | ns | - | ns | <0.001 | <0.001 | ns | ns | ns | ns | ns |
|  | CFT073 (Clinical ExPEC) | | ns | ns | - | <0.001 | <0.001 | <0.001 | 0.002 | 0.02 | <0.001 | <0.001 |
| Naturalized Wastewater *E. coli* Strains | WW10 | | <0.001 | <0.001 | <0.001 | - | Ns | <0.001 | <0.001 | <0.001 | <0.001 | <0.001 |
|  | WW69 | | <0.001 | <0.001 | <0.001 | ns | - | <0.001 | <0.001 | <0.001 | <0.001 | <0.001 |
| Wastewater ExPEC  Strains | WU1036 | | 0.002 | ns | <0.001 | <0.001 | <0.001 | - | ns | ns | ns | ns |
|  | WU664 | | 0.03 | ns | 0.002 | <0.001 | <0.001 | ns | - | ns | ns | ns |
|  | 4B8 | | ns | ns | 0.02 | <0.001 | <0.001 | ns | ns | - | ns | ns |
|  | 2F5 | | 0.006 | ns | <0.001 | <0.001 | <0.001 | ns | ns | ns | - | ns |
|  | 3C4 | | 0.004 | ns | <0.001 | <0.001 | <0.001 | ns | ns | ns | ns | - |

^a^ ns – Not Significant (*p* >0.05)
