# Supplementary material for: Hypo-osmotic stress is an anticipatory trigger of heat-resistance in presumptive extraintestinal pathogenic Escherichia coli isolated from treated sewage
Source: Front Microbiol. 2025 Oct 8;16:1676613. doi: 10.3389/fmicb.2025.1676613 (PMC12540446; doi:10.3389/fmicb.2025.1676613)
Supplement: Supplementary file 5 [file Table_4.docx]

**Supplemental Table S4.** Sequence types and serotypes

| **Strain** | **Sequence Type** ^a^ | **Serotype ^b^** |
| --- | --- | --- |
| ATCC25922 | ST73 | O6:H1 |
| MG1655 | ST10 | O16:H48 |
| CFT073 | ST73 | O6:H1 |
| WW10 | ST635 | O11:H25 |
| WW69 | ST635 | O11:H25 |
| WU1036 | ST131 | O25:H4 |
| WU664 | ST538 | O13/O135:H4 |
| 4B8 | ST131 | O16:H5 |
| 2F5 | ST131 | O25:H4 |
| 3C4 | ST131 | O16:H5 |

^a^ Multilocus sequence typing (MLST) was performed using mlst 2.23.0 (https://github.com/tseemann/mlst) with the *Escherichia coli* #1 scheme.

^b^ Serotyping was done using ECTyper 1.0 (https://github.com/phac-nml/ecoli_serotyping)
